# Complex motivated behaviors for natural rewards following a binge-like regimen of morphine administration: mixed phenotypes of anhedonia and craving after short-term withdrawal

**DOI:** 10.3389/fnbeh.2014.00023

**Published:** 2014-02-03

**Authors:** Yunjing Bai, Yingying Li, Yaodi Lv, Zhengkui Liu, Xigeng Zheng

**Affiliations:** ^1^Key Laboratory of Mental Health, Institute of Psychology, Chinese Academy of SciencesBeijing, China; ^2^University of Chinese Academy of SciencesBeijing, China

**Keywords:** anhedonia, morphine withdrawal, sucrose, sexual reward, social reward, consummatory behavior, operant behavior, appetitive behavior

## Abstract

The anhedonia-like behaviors following about 1-week withdrawal from morphine were examined in the present study. Male rats were pretreated with either a binge-like morphine paradigm or daily saline injection for 5 days. Three types of natural reward were used, food reward (2.5, 4, 15, 30, 40, and 60% sucrose solutions), social reward (male rat) and sexual reward (estrous female rat). For each type of natural stimulus, consummatory behavior and motivational behaviors under varied testing conditions were investigated. The results showed that the morphine-treated rats significantly reduced their consumption of 2.5% sucrose solution during the 1-h consumption testing and their operant responding for 15, 30, and 40% sucrose solutions under a fixed ratio 1 (FR1) schedule. However, performance under a progressive ratio (PR) schedule increased in morphine-treated rats reinforced with 60% sucrose solution, but not in those reinforced with sucrose concentrations lower than 60%. Pretreatment with morphine significantly decreased the male rats' ejaculation frequency (EF) during the 1-h copulation testing, and impaired the maintenance of appetitive motivations to sexual and social stimuli under a free-approach condition. Moreover, the morphine-treated rats demonstrated a diminished motivation to approach social stimulus in the effort-based appetitive behavior test but showed a remarkable increase in motivation to approach sexual stimulus in the risky appetitive behavior test. These results demonstrated some complex motivated behaviors following about 1 week of morphine withdrawal: (1) The anhedonia-like behavior was consistently found in animals withdrawn from morphine. However, for a given reward, there was often a dissociation of the consummatory behaviors from the motivational behaviors, and whether the consummatory or the motivational anhedonia-like behaviors could be discovered heavily depended on the type and magnitude of the reward and the type of testing task; (2) These anhedonia-like behaviors coexisted with a craving for the high-incentive reward which was evidenced by the increased PR performance for the 60% sucrose solution and the heightened risky appetitive behavior for the sexual stimulus. The craving for the high-incentive reward alongside with the impaired inhibitory control in drug-withdrawn subjects might form one of psychological mechanisms underlying drug relapse after withdrawal.

## Introduction

Considerable evidence indicates that withdrawal from repeated exposure to drugs of abuse leads to a series of affective responses, such as anhedonia, dysphoria, irritability, etc. These negative emotional states, which result from dysregulation of brain reward system, are hypothesized to contribute to compulsive drug seeking behavior and relapse to drug use even after protracted abstinence (Koob, 2008, 2009). Among these affective withdrawal effects, anhedonia, defined as diminished interest or pleasure in response to rewarding stimuli (APA, 2000), is a core feature of reward deficits and has become an important subject of clinical and preclinical studies (Der-Avakian and Markou, 2012). Now it has been clear that anhedonia not only reflects deficits in hedonic capacity (the capacity to feel pleasure), but also is closely linked to the reward processes including reward evaluation, motivation to seek rewards and decision-making (Der-Avakian and Markou, 2012). In a recent review, Treadway and his colleague suggested that anhedonia could be redefined with “consummatory anhedonia” and “motivational anhedonia” in view of the distinction between deficits in the hedonic response to rewards and a diminished motivation to pursue rewards (Treadway and Zald, 2011). These opinions provide a theoretical framework for assessment of anhedonia-like behaviors following withdrawal from drugs of abuse.

The natural rewards, food, sex, and social reward, have been used to examine the anhedonia-like behaviors following drug withdrawal. Usually, only one type of the natural reward such as food or sexual reward was adopted in the majority of preclinical literatures. For example, the consumptive behaviors for sucrose/sweet pellet and operant responding on a FR or PR schedule of reinforcement have been evaluated as behavioral measures of anhedonia (Lieblich et al., 1991; Barr and Phillips, 1999; Hellemans et al., 2002; Russig et al., 2003; Lesage et al., 2006; Zhang et al., 2007; Cooper et al., 2010; Der-Avakian and Markou, 2010; Galaj et al., 2013). In the experiments performed with social and/or sexual rewards, the effects of drug withdrawal on copulatory behavior or appetitive motivation for the rewarding stimulus (free approach or conditioned approach) have been assessed (Ferrari and Giuliani, 1997; Barr et al., 1999; Fiorino and Phillips, 1999; Nocjar and Panksepp, 2002, 2007; Cui et al., 2004). However, there exist large inconsistencies between the results derived from the previous preclinical literatures. This is most probably due to the adoption of diverged magnitudes of the reward (sucrose/sweet pellet), different testing tasks as well as different withdrawal periods in those studies. Therefore, it seems still difficult to find a regular pattern of anhedonia-like states associated with drug withdrawal.

In the present study, aiming to portrait a multidimensional profile for the anhedonia-like or sensitization-like responses to natural stimuli during morphine withdrawal, we presented varied testing tasks which mainly fell into consummatory vs. motivational domain. In each domain, we used at least two of the following natural rewarding stimuli, i.e., sucrose solution (with a range of concentrations), social stimulus (male rat) as well as sexual stimulus (estrous female rat) for comparisons. Besides, within the motivational domain, another behavioral construct, the cost/benefit computation was also involved, i.e., the FR1 (fixed ratio 1) vs. PR (progressive ratio) procedure in the testing of operant responding for sucrose as well as the free approach vs. decisional approach in the social and sexual appetitive motivation tests. The behavioral procedures for decisional approach were firstly established in our laboratory to imitate the PR procedure for operant behaviors. Although both the FR and PR schedules of reinforcement have been used to assess the reinforcing efficacy of a given reward and the motivation to obtain the reward, some disparities between the results from these two procedures have been illustrated, and the results are probably determined by the work requirement of a procedure (Arnold and Roberts, 1997; Salamone et al., 2007). Here, compared to the FR1 response as well as free approach (low-effort performance), the animals tested under the procedures of PR performance and decisional approach had to exert a progressively increasing amount of effort to obtain each food reward or approach sexual/social stimulus. Accordingly, the increased PR performance is more frequently considered as a measure of high motivation to obtain the food or drug reward (Deroche-Gamonet et al., 2004; de Jong et al., 2012), and is evidenced to correlate with a state of craving for food reward in human participants (Willner et al., 1998).

We adopted a chronic, binge-like morphine treatment paradigm since repeated opioid treatment can result in dependence and the addictive state that persists for a long time after cessation of the exposure (Nestler and Aghajanian, 1997), and a similar morphine treatment paradigm has been demonstrated to induce intracellular changes in opioid receptors and G proteins (Fabian et al., 2002). In the present study, all the testing tasks were carried out following about 1-week withdrawal from morphine in order to avoid the aversive effects of acute withdrawal. Moreover, the anhedonia-like behaviors seem not easy to be definitely or consistently found after acute withdrawal, as demonstrated by various results from different studies (Barr and Phillips, 1999; Barr et al., 1999; Lesage et al., 2006; Nocjar and Panksepp, 2007; Zhang et al., 2007; Der-Avakian and Markou, 2010) and the fact that only some of the elevations in reward threshold (measured by intracranial self-stimulation) during acute withdrawal can last for up to 1 week (Koob, 2009). We proposed that, in the present study with multiple natural rewards and varied testing tasks, more meaningful results would be obtained, which could evidence that differentiated behavioral construct might have made decisive contributions to the qualitatively different or even completely opposite phenotypic expressions.

## Materials and methods

### Animals and housing

Male and female Sprague-Dawley rats (Vital River Animal Center, Beijing, China) were housed in colony rooms with a controlled temperature (22–26°C) and a 12 h/12 h light/dark cycle. All rats were allowed to habituate to the housing for at least 5 days with food and water available *ad libitum*, and were gently handled daily for at least 3 days prior to the beginning of experiments. This study is approved by the International Review Board (IRB) of the Institute of Psychology, Chinese Academy of Sciences, and all experiments were conducted in accordance with the National Institutes of Health Guide for Care and Use of Laboratory Animals (Publication No.85-23, revised 1985).

### The binge-like morphine pretreatment

The male rats were pretreated with either a binge-like regimen of morphine administration or saline according to the different group assignments. Morphine hydrochloride (Qinghai Pharmaceutical Co. Ltd, Qinghai, China)/saline were intraperitoneally (i.p.) administered twice daily for 5 days. The escalating-dose morphine injection was as follow: 10, 20, 20, 40, 40, 40, 40, 40, 40, 40 mg/kg. Two doses of morphine administered on each day were separated by a 6 h interval at least. The rats were returned to their home cages immediately after each injection. All of the animals were weighed every day throughout the pretreatment and 7-days withdrawal.

### Withdrawal symptoms assessment

After the termination of the injection schedule, withdrawal symptoms assessment commenced 22–24 h for the animals, a time point marked by opioid withdrawal. Beginning on the day after the last injection and continuing at daily intervals for 5 days, rats were placed in a transparent glass cylinder (diameter = 30 cm, high = 60 cm) and tested for signs of opiate withdrawal for 1 h. Withdrawal severity was assessed according to a slightly modified rating scale described elsewhere (Cicero et al., 2002). The signs and symptoms and their weighting factors were shown in Table 1. These observations took place for a 60-min period on each testing day.

**Table 1:** **Graded and checked signs of withdrawal**.

**Sign**	**Weighting factor**
**GRADED SIGNS**	
Body weight loss (every 24 h)	1 for each 1% body weight loss
**WET-DOG SHAKES**	
1–2	2
3–10	4
10+	6
**CHECKED SIGNS**	
Teeth chattering	2
Abnormal posture	3
Ptosis	2

### Experiment 1 the consummatory and operant behaviors for sucrose solutions after withdrawal from morphine

#### Animals

Two hundred and seventeen Sprague-Dawley rats weighing 250–270 g on arrival, were housed individually in the home cages (25 × 22.5 × 30 cm) under a 12-h light-dark cycle (lights on at 0700). Eighty animals were used to perform the consummatory behaviors and the other 72 animals were presented to operant testing.

#### Apparatus

Consummatory behavioral (i.e., sucrose consumption) sessions were conducted in the home cages; one or two plastic 250-ml graduated drinking bottles were mounted on each cage with rubber covered clamps. Drinking tubes were fitted to the cylinders. Operant experiments were carried out in 8 operant chambers (AniLab Software and Instruments Co., Ltd. Ningbo, China) enclosed in sound-attenuating boxes. Each chamber was fitted with two nose-poke operandi, one located on each side of a central liquid receptacle. Two yellow LED cue lights (20 mW) were separately situated inside each nose-poke. A white cage light was allocated 20 cm above the right nose-poke. Solution was delivered through a metal spout attached to a 60-ml syringe pump with tubing that delivered fluid at a speed of 34.450 ml/min. Pumps were calibrated to dispense 0.08 ml (0.139 s) of solution into a liquid receptacle per reinforcement. There was a time-out period of 10 s following each reinforcement and subsequent responding produced no effect during this period.

#### Familiarization of sucrose solutions

All rats were familiarized with sucrose to avoid neophobia by giving them 48 h access to a 2.5% sucrose solution (w/v) in addition to water and food in their home cages (Monteggia et al., 2007). Food was given *ad libitum* during this period. Bottle weight was recorded prior to, and immediately after the test. Sucrose intake was calculated as a function of body weight, with the amount (g) of solution consumed per weight (100 g). Preference for sucrose, determined by the ratio of sucrose solution to total solution intake, was expressed as percentage of the total intake. Then the rats were evenly assigned into either the saline-pretreatment group (*n* = 111) or the morphine-pretreatment group (*n* = 106).

#### Sucrose consumption testing

A total of 112 rats (including 56 morphine-pretreated rats and 56 saline-pretreated rats) were used in a 1-h sucrose consumption testing (Barr and Phillips, 1999). The testing was conducted in the home cages on day 8 of withdrawal. Before the testing, rats were placed in a 23-h food deprivation schedule and water was removed for 2 h before the testing. The animals were assigned into 14 groups (8 rats/group), i.e., Morphine-0%, Saline-0%, Morphine-2.5%, Saline-2.5%, Morphine-4%, Saline-4%, Morphine-15%, Saline-15%, Morphine-30%, Saline-30%, Morphine-40%, Saline-40%, Morphine-60%, and Saline-60%. Thus, rats were presented with a single bottle contained either 0%, 2.5%, 4%, 15%, 30%, 40%, or 60% sucrose solution for free consumption. Bottle weight was recorded prior to, and immediately after the testing. After the testing, the rats were given *ad libitum* access to lab chow and water. The consumption testing was conducted in the dark cycle, since rats presented higher activity during night.

#### Operant procedures

The other 105 rats were assigned into 12 groups, i.e., Morphine-2.5% (*n* = 8), Saline-2.5% (*n* = 9), Morphine-4% (*n* = 8), Saline-4% (*n* = 9), Morphine-15% (*n* = 9), Saline-15% (*n* = 10), Morphine-30% (*n* = 9), Saline-30% (*n* = 10), Morphine-40% (*n* = 9), Saline-40% (*n* = 9), Morphine-60% (*n* = 7), Saline-60% (*n* = 8). On day 4 of withdrawal, those rats were habituated to the operant chamber for 15 min, and then were immediately given a 60-min session to be trained to associate stimuli with the delivery of 0.08 ml liquid dispensed in the central liquid receptacle. In the 60-min session, the chamber was illuminated by two nose-poke lights, and liquid was delivered into the central liquid receptacle on a variable interval (VI) 40-s schedule (Chudasama and Robbins, 2003). One second before a liquid dropped, the house light (as the stimuli) was switched and remained on for a total of 4 During the habituation, tap water was used as the liquid reward. To ensure that the rats drink more water in the operating chambers, water and food was removed from their house cages 12 and 18 h before the habituation started, respectively. Throughout the operant training and testing, the rats were allowed to have access to food during 1 h per day after each daily session in order to maintain a body weight above 85% percent of their baseline weight (Dias-Ferreira et al., 2009).

On the day after habituation, animals were trained to attain liquid under a FR1 schedule of reinforcement (i.e., one nose poke resulted in a drop of 0.08 ml of either 2.5, 4, 15, 30, 40, or 60% sucrose solution, according to which group they belonged to) for 2 days (Rossetti et al., 2013). During the illumination of the active nose-poke light, introduction of the animal's nose into the correct nose-poke hole (active device) would turn on the house light and then, 1 s later, switched on the syringe pump. Nose pokes in the other nose-poke hole (inactive device) had no scheduled consequences. The acquisition criterion was defined as that a rat attained 60 reinforcers within 40 min, but if it failed, it would be sent back to the home cage and started an added session in 2–3 h. Each rat could only be trained at most twice daily. Animals that did not reach the acquisition criterion for 3 times were deleted. Water was removed for 2 h before each daily session throughout the operant training and testing, and food was supplied after the session completed for 1 h (Dias-Ferreira et al., 2009).

Following the FR 1 testing (i.e., withdrawal day 8), rats were subjected to a 3-days progressive ratio (PR) schedule for 1 h whereby successive reinforcements could be earned according to the following number of nose pokes: 1, 2, 4, 6, 9, 12, 15, 20, 25, 32, 40, 50, 62, 77, 95, 118, 145… (Brennan et al., 2001). The final ratio achieved represented the “breaking point” value, which was represented as “reinforcers obtained” in the figures. The session ended either when rats failed to reach the next nose poke criterion within 30 min, or when the session duration reached 1 h. All operating training and testing took place during the light phase, and each animal received the same concentration of sucrose solution throughout the study (i.e., from consumption testing, FR1 testing to PR testing). Percentage of nose pokes in the inactive hole was calculated by dividing the number of total nose pokes (both in the active and inactive holes).

#### Data analysis

The body weight of rats and scores of withdrawal symptoms were analyzed by Two-Way repeated measures' analysis of variance (ANOVA) with “day” as the within-subjects factor, “pretreatment” as the between-subjects factors. For free solution consumption, reinforcers obtained in the FR 1, data were analyzed using a Two-Way ANOVA, with “pretreatment” and “concentration” as the between-subjects factors. For reinforcers obtained in the temporal sequence during the 1-h FR1 session and 3-day PR tests, a Two-Way repeated measures' ANOVA was used, with “time” or “day,” respectively, as the within-subjects factor, “pretreatment” as the between-subjects factor. In case of significant interaction, analysis of simple effects was performed. A two-tailed significance level 0.05 was used. All statistical analyses were performed using SPSS Statistics (version 16.0; SPSS Inc., Chicago, IL, USA).

### Experiment 2 the consummatory and appetitive behaviors for social and sexual rewards after withdrawal from morphine

#### Animals

Sprague-Dawley rats were housed four per cage (50 × 22.5 × 30 cm) in a colony room with a reversed 12 h/12 h light/dark cycle (lights on at 21:00). Males weighed 330–400 g at the beginning of the experiments and females weighed 230–250 g upon ovariectomy. Females were bilaterally ovariectomized under 1% pentobarbital sodium (55 mg/kg, i.p.) anesthesia at least 2 weeks before use. Artificial estrus was induced by subcutaneous treatment with estradiol benzoate (25 μg/rat) and progesterone (1 mg/rat) about 48–52 h and 4–6 h before tests, respectively. All tests were performed between 10:00 and 20:00 during the dark phase of the cycle.

#### Screening male rats

Male rats were prescreened before all the experiments, and only those that performed ejaculation within 30 min for 3 consecutive days were used. Screening was conducted under dim light during the dark phase of the light/dark cycle. Individual male rats were placed in a carton box (60 × 50 × 40 cm height) with pine wood shaving bedding and allowed a 5-min adaptation period. A receptive female rat was then introduced and male copulatory behaviors were monitored by experienced observers. The copulation on each day ended after the rat completed its first ejaculation within 30 min. The male rats that passed the screening were assigned to the saline or the morphine pretreatment group.

#### Apparatus

Four Open-field reward-proximity chambers made of black Plexiglas were used for assessment of the simple and decisional appetitive behaviors for social or sexual reward. Each open-field arena (85 × 35 × 50 cm high) had a wire-screen stimulus-cage (15 × 25 × 25 cm high) which was mounted at one end. The front of the cage was made of wire mesh (1-mm wire, mesh size 10 × 10 mm), which allowed the subjects to approach and investigate (i.e., sniff) the animals (male or estrous female rat) in the cage but prevented reward consumption. The open-field arena was transected into three compartments by two partitions, and the two larger end compartments, equal-sized (35 × 35 × 50 cm high), were used as copulatory chambers and allowed assessment of consumptive sexual behavior (male copulation).

#### Simple appetitive behavior (free approach) testing

**Figure 1:**
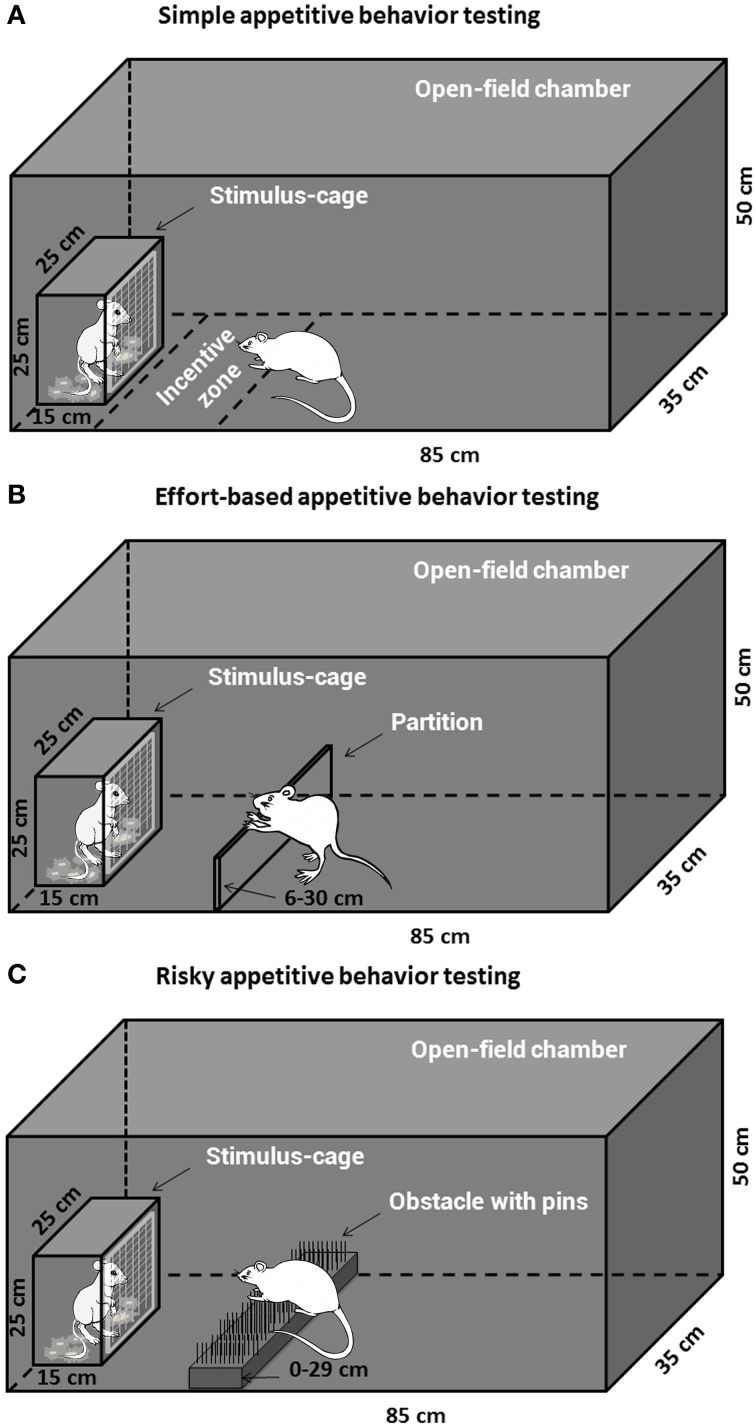
**The apparatus for the social or sexual appetitive behavior testing in male rats**. The open-field chamber with a stimulus-cage holding a male or estrous female rat could be adapted to different appetitive behavior testing tasks. **(A)** The subjects could freely approach and investigate the incentive rat inside the stimulus-cage during the simple appetitive behavior testing. **(B)** The subjects had to expend labors, i.e., climb over a continuously heightened partition, to approach the stimulus-cage during the effort-based appetitive behavior testing. **(C)** The subjects had to surmount a dangerous obstacle, i.e., climb over a continuously heightened board thick with pins, to approach the stimulus-cage during the risky appetitive behavior testing.

Twenty-nine animals pretreated with either morphine or saline were assigned to the testing of appetitive motivation for social and sexual rewards, i.e., Saline-male (*n* = 7), Morphine-male (*n* = 7), Saline-female (*n* = 7), Morphine-female (*n* = 8). The apparatus for the testing was shown in Figure 1A. On the day before the appetitive behavior testing, all the subjects were habituated for 30 min in the open-field arena. On the testing day (withdrawal day 7), after habituation for 10 min in the open-field arena, a male rat or a sexually receptive female rat was placed in the stimulus-cage and the behavior of each subject was videotaped for 1 h by a camera and later analyzed using EthoVision software XT 7.1 (Noldus, The Netherlands). The open-field and stimulus-cage were wiped clean with 0.1% acetic acid in water between subjects to eliminate olfactory cues. As the measures of social interest or sexual incentive motivation, for each subject, the time spent on sniffing the stimulus-cage (sniffing time, i.e., nose-point within the wire-screen) and the time spent in the incentive zone (35 × 20 cm) adjoined to the stimulus-cage (zone time, i.e., center-point in the incentive zone) were automatically collected by the software.

#### Male copulation

About 2–4 h after the simple appetitive behavior testing, the male copulation assessment was performed. A male subject was placed into the copulatory chamber and 5 min later, a sexually receptive female rat was introduced. The sexual behavior was videotaped for 1 h and subsequently scored for standard measures of male copulatory behavior by an observer that was blind to the drug history of each animal.

#### Decisional appetitive behavior (decisional approach) testing

***Effort-based appetitive behavior testing***. The animals pretreated with either drug or saline were assigned to the testing of effort-based appetitive behavior for social or sexual reward, i.e., Saline-male (n = 10), Morphine-male (n = 9), Saline-female (n = 11), Morphine-female (n = 9). The apparatus for the testing was displayed in Figure 1B. On the day before the testing (withdrawal day 6), all subjects were habituated for 15 min in the open-field arena with a 6 cm-high, transparent, Plexiglas partition which was installed about 20 cm in front of the wire-screen of stimulus-cage. On the testing day (withdrawal day 7), after habituation for 10 min in the open-field arena (with a 6 cm-high partition), a male rat or a sexually receptive female rat was placed in the stimulus-cage to allow the subject to freely approach and investigate the incentive rat for 5 min. Moreover, some clean bedding (about 30 g) had been placed on the floor of stimulus-cage containing male rat, while the female-soiled bedding was placed on the floor of stimulus-cage containing female rat. After 5 min-freely approaching, the subject was moved away from the stimulus-cage, and the test began with adding one 2 cm-high lath (transparent, Plexiglas) on the already existing 6 cm-high partition, i.e., 8 cm-high partition. During the following part of the test, the partition was constantly heightened by repeatedly building with 2 cm-high laths. The test contained 12 trials. One trial was finished when the subject climbed over the partition to approach the reward 3 times within 4 min, and the next trial was started. The subject was moved away from the stimulus-cage about 15–20 s after climbing over the partition every time. Every trial began with adding a 2 cm-high lath on the existing partition. Thus, the heights of partition from trial 1 to trial 12 were as follows: 8, 10, 12, 14, 16, 18, 20, 22, 24, 26, 28, and 30 cm. If the subject climbed over the partition less than 3 times within 4 min, the test ended and the maximum height of the partition that the subject had climbed over to approach the stimulus cage was recorded as an index of appetitive motivation. The female-soiled bedding was collected from one cage that had contained three sexually receptive females during 5 days (replace females every day) and stored in the freezer until the day of the experiment. The social and sexual motivation tests were always performed in the two separate chambers in order to avoid cross contamination between distinct olfactory stimuli.

***Risky appetitive behavior testing***. The rats pretreated with either morphine or saline were assigned to the testing of risky appetitive behavior for social or sexual reward, i.e., Saline-male (n = 12), Morphine-male (n = 12), Saline-female (n = 16), Morphine-female (n = 15). The apparatus for the testing was displayed in Figure 1C. On the day before the testing, all subjects were habituated for 15 min in the open-field arena (without any obstacle). On the testing day (withdrawal day 7), after habituation for 10 min in the open-field arena (without any obstacle), a male rat or a sexually receptive female rat was placed in the stimulus-cage to allow the subject to freely approach and investigate the incentive rat for 5 min. Moreover, some clean bedding (about 30 g) had been placed on the floor of stimulus-cage containing the male rat, while the female-soiled bedding was placed on the floor of stimulus-cage containing the female rat. After 5 min-free approach, the subject was moved away from the stimulus-cage, and the test began with an obstacle, a 14 cm-wide board thick with pins, being installed on the floor about 20 cm in front of the wire-screen of stimulus-cage. With the test continuing, the obstacle became more and more difficult to surmount by means of replacing the board thick with pins in different styles and repeatedly heightening the board. According to length of the pins and average distance between pins, three types of board were used: (a) Length—0.5 cm, average distance—1 cm; (b) Length—0.8 cm, average distance—0.5 cm; (c) Length—2 cm, average distance—1 cm. The board was repeatedly heightened as follows: 0, 2, 4, 7, 10, 13, 17, 21, 25, 29 cm. Thus, the 12-levels difficulties of surmounting the obstacle, i.e., 12 trials during the test were as follows: a + 0 cm, a + 2 cm, a + 4 cm, b + 4 cm, b + 7 cm, b + 10 cm, b + 13 cm, b + 17 cm, c + 17 cm, c + 21 cm, c + 25 cm, c + 29 cm. One trial was finished when the subject climbed or jumped over the obstacle 3 times within 4 min, and then the next trial was started. The subject was moved away from the stimulus-cage about 15–20 s after surmounting the obstacle every time. If the subject surmounted the obstacle less than 3 times within 4 min, the test ended. The amount of difficulty the subject conquered every time to approach the stimulus-cage was graded and summed up to the total score for each subject Table 2.

**Table 2:** **Risky appetitive behavior testing**.

**Trial**	**Amount of difficulty**	**Graded per approach**
1	a + 0 cm	0.5
2	a + 2 cm	1.0
3	a + 4 cm	1.5
4	b + 4 cm	3.0
5	b + 7 cm	3.5
6	b + 10 cm	4.0
7	b + 13 cm	4.5
8	b + 17 cm	5.0
9	c + 17 cm	6.0
10	c + 21 cm	6.5
11	c + 25 cm	7.0
12	c + 29 cm	7.5

#### Data analysis

The sniffing time and zone time spent on social or sexual stimulus were analyzed by Two-Way repeated-measures analysis of variance (ANOVA), with “time” as the within-subjects factor and “pretreatment” as the between-subjects factors. For the decisional appetitive behaviors, data were analyzed by Two-Way ANOVA, with “pretreatment” and “reward” as the between-subjects factors. In cases of significant interactions, analyses of simple effects were performed. The measures of male copulatory behavior were analyzed using *t*-tests. A two-tailed significance level of 0.05 was used. All statistical analyses were performed using SPSS Statistics (version 16.0; SPSS Inc., Chicago, IL, USA).

## Results

### Withdrawal symptoms

**Figure 2:**
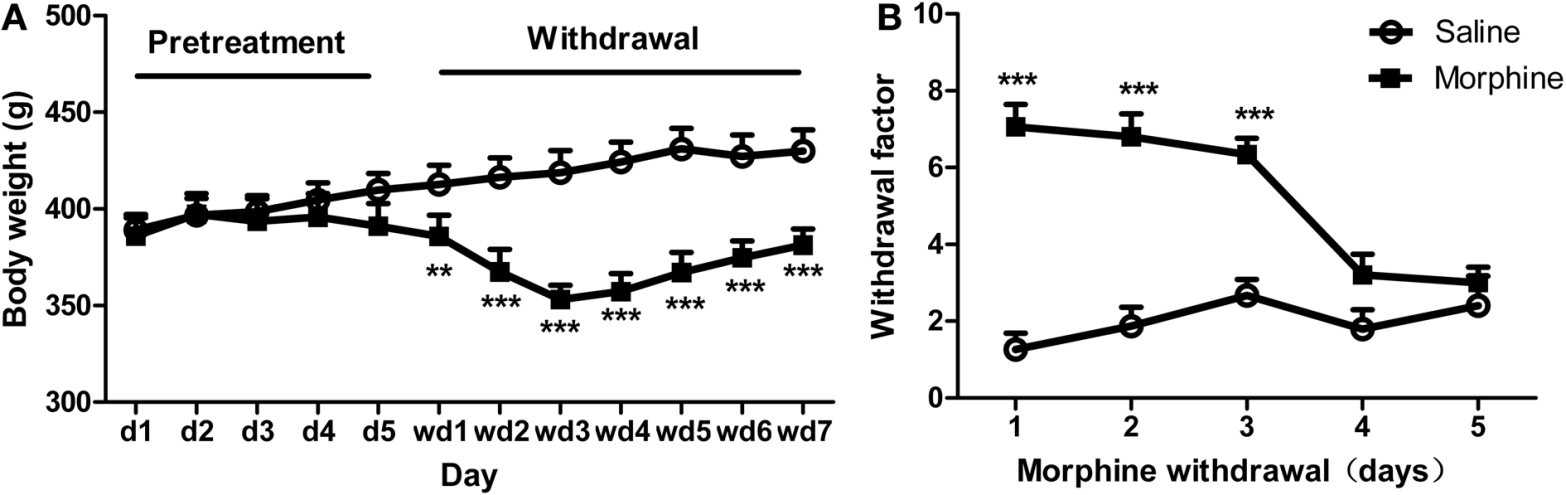
**Other physical and behavioral signs after morphine exposure. (A)** Body weights of the rats throughout the pretreatment and withdrawal. **(B)** Withdrawal scores in rats following the cessation of daily morphine or saline injections. ^**^P < 0.01, ^***^P < 0.001, relative to saline animals. Values are mean ± s.e.m.

The body weights of all the rats increased equally during the early phase of pretreatments (Figure 2A). However, after ceasing morphine administration, a Two-Way repeated ANOVA showed a significant “pretreatment” effect [*F*_(1, 39)_ = 33.374, *P* < 0.001], as well as significant effects of “day” [*F*_(6, 234)_ = 53.632, *P* < 0.001] and pretreatment × day [*F*_(6, 234)_ = 54.531, *P* < 0.001]. Analysis of simple effects revealed a significant difference between morphine and saline groups after the termination of the injection, i.e., on day 1 [*F*_(1, 39)_ = 9.41, *P* < 0.01], day 2 [*F*_(1, 39)_ = 31.13, *P* < 0.001], day 3 [*F*_(1, 39)_ = 56.10, *P* < 0.001], day 4 [*F*_(1, 39)_ = 52.32, *P* < 0.001], day 5 [*F*_(1, 39)_ = 39.79, *P* < 0.001], day 6 [*F*_(1, 39)_ = 27.88, *P* < 0.001], and day 7 [*F*_(1, 39)_ = 24.98, *P* < 0.001]. Figure 2B shows the weighted withdrawal factor. A significant interaction was found between “pretreatment” and “day” [*F*_(4, 112)_ = 12.138, *P* < 0.001], with significant differences between groups on day 1 [*F*_(1, 28)_ = 65.49, *P* < 0.001], day 2 [*F*_(1, 28)_ = 40.52, *P* < 0.001], and day 3 [*F*_(1, 28)_ = 36.83, *P* < 0.001] after withdrawal. Accordingly, the regimen of drug pretreatment is sufficient to produce severe physical dependence.

### Effect of withdrawal on sucrose consumption

**Figure 3:**
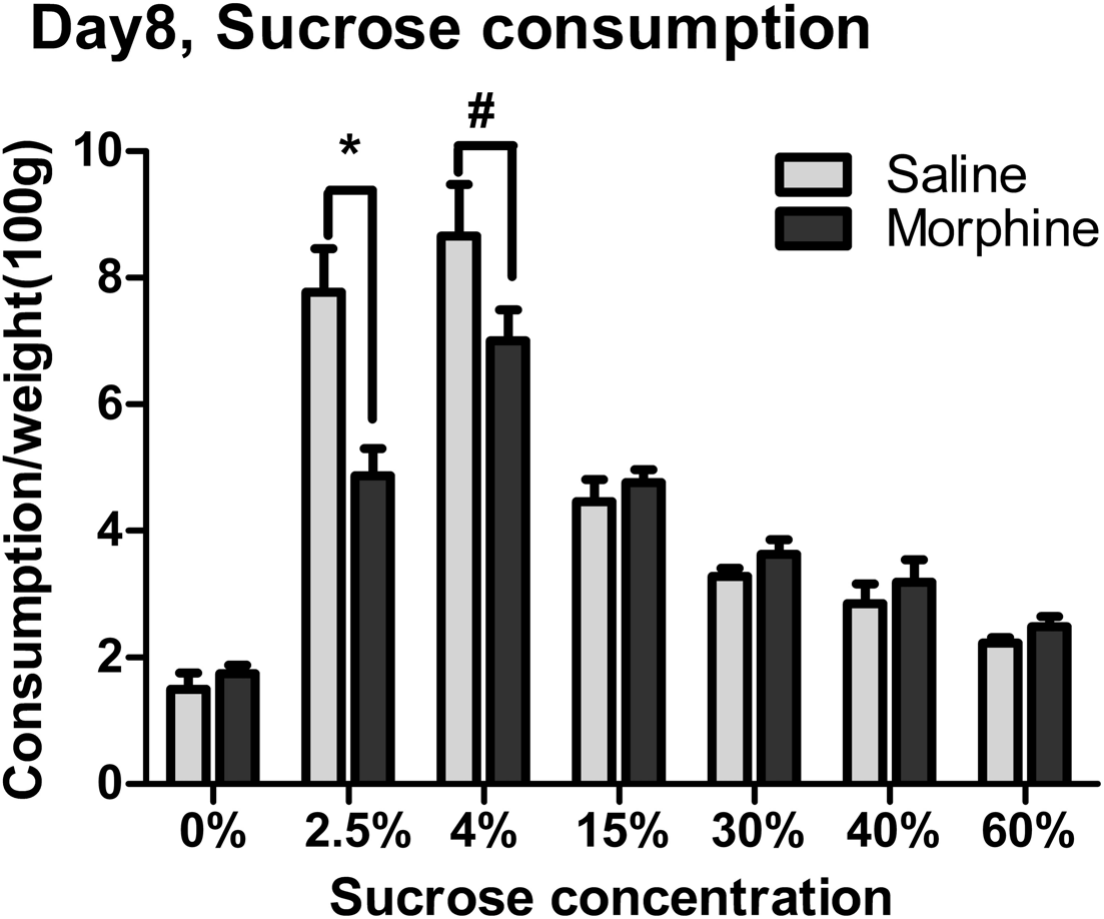
**The effects of morphine withdrawal on free consumption of varying concentrations of sucrose solution from a single bottle**. Sucrose consumption in 1-h session was assessed under food-deprivation on day 8 of withdrawal. ^*^P < 0.05, #0.05 < *P* < 0.01, relative to saline group tested with the same concentration of sucrose solution. Values are mean ± s.e.m.

Sucrose consumption on day 8 of morphine withdrawal is shown in Figure 3. Two-Way ANOVA revealed that there was a significant main effect of “pretreatment” [*F*_(1, 97)_ = 6.143, *P* < 0.05], “concentration” [*F*_(6, 97)_ = 65.260, *P* < 0.001], and a significant interaction between “pretreatment” and “concentration” [*F*_(6, 97)_ = 6.446, *P* < 0.001]. Analysis of simple effects revealed a significant difference between morphine and saline groups when the solution concentration was 2.5% [*F*_(1, 97)_ = 6.13, *P* < 0.05], as well as a marginal significance between Morphine-4% and Saline-4% groups [*F*_(1, 97)_ = 3.07, *P* = 0.083].

### Effect of withdrawal on operant responses for sucrose

**Figure 4:**
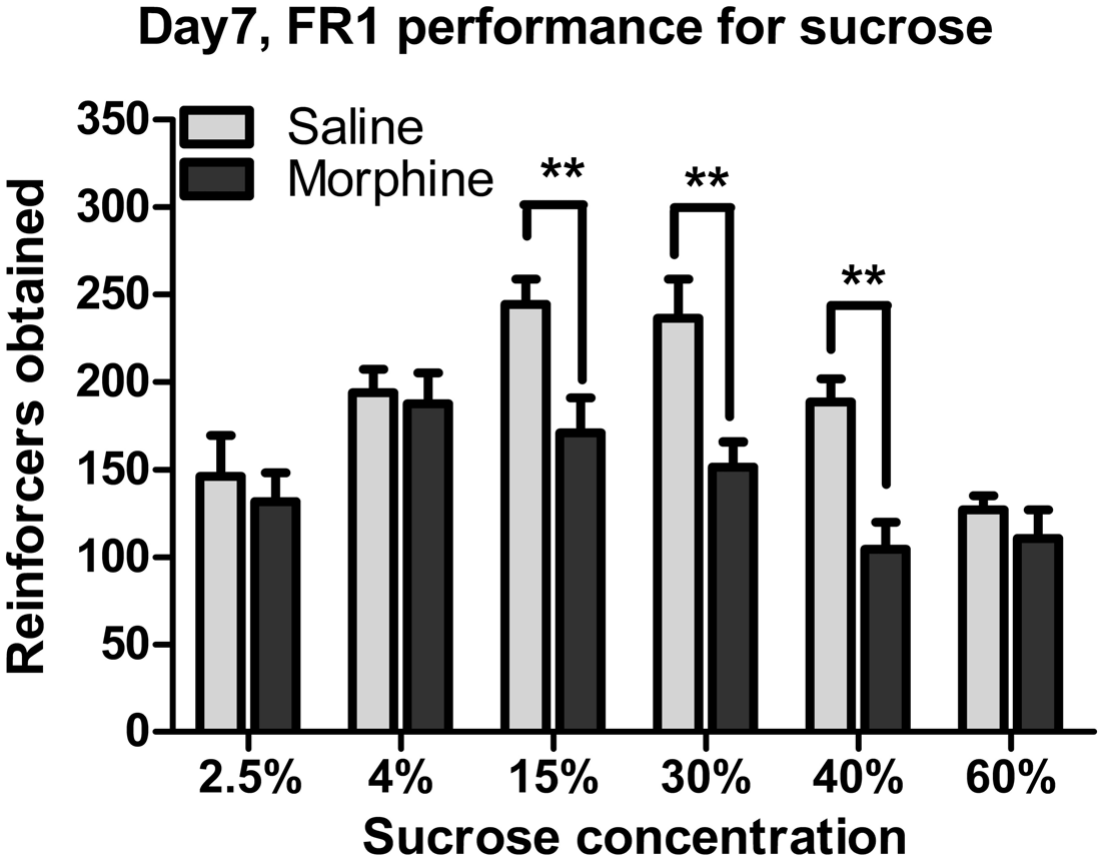
**Number of reinforcers obtained under a FR1 schedule of reinforcement on day 7 of withdrawal**. ^**^P < 0.01, relative to saline group tested with the same concentration of sucrose solution. Values are mean ± s.e.m.

**Figure 5:**
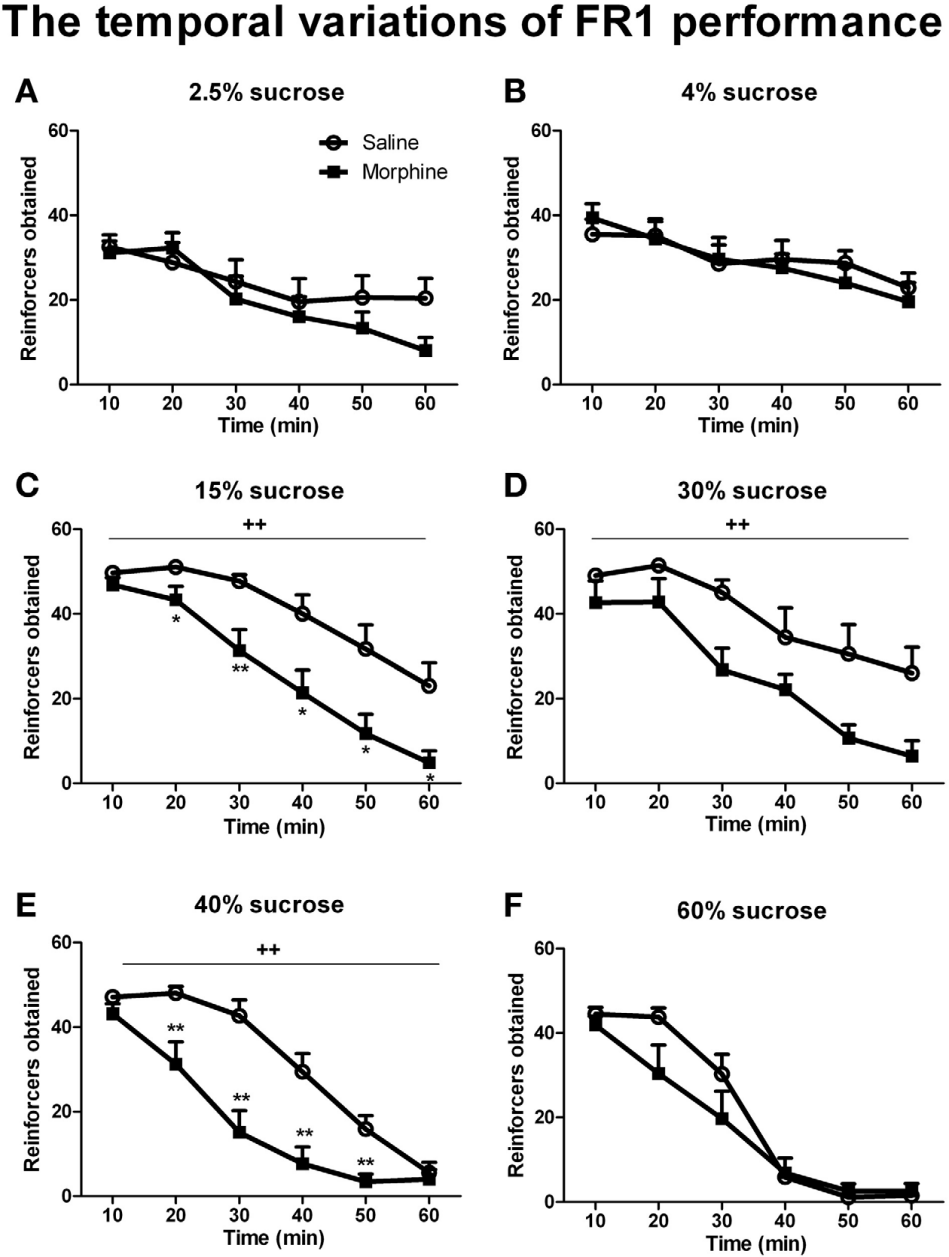
**Number of reinforcers obtained across the 1-h FR1 session (10 min/block)**. Rats were reinforced by sucrose solutions with different concentrations of 2.5% **(A)**, 4% **(B)**, 15%**(C)**, 30% **(D)**, 40%**(E)**, or 60% **(F)**. Values represent the solution delivered. ^*^P < 0.05, ^**^P < 0.01, relative to saline group tested with the same concentration of sucrose solution. ^++^*P* < 0.01, a significant main effect of pretreatment. Values are mean ± s.e.m.

**Figure 6:**
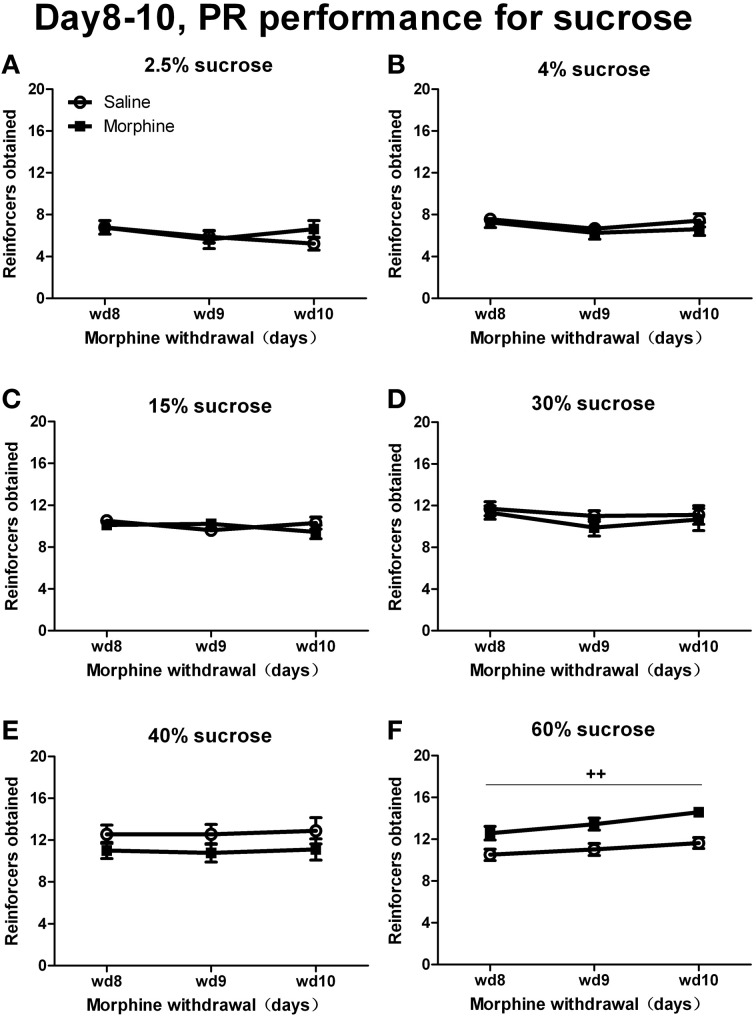
**Number of reinforcers obtained under a PR schedule of reinforcement on day 8–10 of withdrawal**. Rats were reinforced by sucrose solutions with different concentrations of 2.5% **(A)**, 4% **(B)**, 15% **(C)**, 30% **(D)**, 40% **(E)**, or 60% **(F)**. ^++^*P* < 0.01, a significant main effect of pretreatment. Values are mean ± s.e.m.

Figures 4, 6 illustrate the number of reinforcers obtained under a FR1 schedule and breaking points under a PR schedule for saline and morphine groups on day 7–10 of drug withdrawal. On day 7 of morphine withdrawal (i.e., the FR1 session), a Two-Way ANOVA indicates that there were significant main effects of “pretreatment” [*F*_(1, 93)_ = 23.096, *P* < 0.001] and “concentration” [*F*_(5, 93)_ = 8.933, *P* < 0.001], and a significant interaction between “pretreatment” and “concentration” [*F*_(5, 93)_ = 2.310, *P* < 0.05]. Analysis of simple-effects showed that morphine groups significantly decreased their responses for 15% [*F*_(1, 93)_ = 7.91, *P* < 0.01], 30% [*F*_(1, 93)_ = 10.13, *P* < 0.01] and 40% [*F*_(1, 93)_ = 8.83, *P* < 0.01] sucrose solutions. Figure 5 demonstrated reinforcers obtained in the temporal sequence during the 1-h FR1 session. For 15 and 40% sucrose solutions, Two-Way repeated measures' ANOVA found a significant effect of “pretreatment” [15% sucrose: *F*_(1, 17)_ = 13.654, *P* < 0.01; 40% sucrose: *F*_(1, 16)_ = 17.451, *P* < 0.01] and “time” [15% sucrose: *F*_(5, 85)_ = 40.900, *P* < 0.001; 40% sucrose: *F*_(1, 80)_ = 76.071, *P* < 0.001], as well as a significant interaction between “pretreatment” and “time” [15% sucrose: *F*_(5, 85)_ = 2.550, *P* < 0.05; 40% sucrose: *F*_(5, 80)_ = 7.355, *P* < 0.001]. Analysis of simple effects revealed morphine groups drank significantly less 15% sucrose and 40% sucrose in the second [15% sucrose: *F*_(1, 85)_ = 6.25, *P* < 0.05; 40% sucrose: *F*_(1, 80)_ = 9.34, *P* < 0.01], the third [15% sucrose: *F*_(1, 85)_ = 10.81, *P* < 0.01; 40% sucrose: *F*_(1, 80)_ = 18.81, *P* < 0.01], the fourth [15% sucrose: *F*_(1, 85)_ = 7.26, *P* < 0.05; 40% sucrose: *F*_(1, 80)_ = 13.95, *P* < 0.01], the fifth [15% sucrose: *F*_(1, 85)_ = 7.19, *P* < 0.05; 40% sucrose: *F*_(1, 80)_ = 11.70, *P* < 0.01] and the last 10-min [only significant when responding for 15% sucrose: *F*_(1, 85)_ = 8.25, *P* < 0.05]. As for the 30% sucrose, data analysis found a significant effect of “pretreatment” [*F*_(1, 17)_ = 9.604, *P* < 0.01] and “time” [*F*_(1, 85)_ = 22.974, *P* < 0.01], but no significant interaction between “pretreatment” and “time” [*F*_(1, 85)_ = 1.190, *P* > 0.05]. For the lower concentration of sucrose solutions (i.e., 2.5 and 4%) and the highest concentration (i.e., 60%), there was a significant effect of “time” [2.5% sucrose: *F*_(5, 75)_ = 11.599, *P* < 0.001; 4% sucrose: *F*_(5, 75)_ = 11.624, *P* < 0.001; 60% sucrose: *F*_(5, 65)_ = 84.467, *P* < 0.001], but no significant effect of “pretreatment” [2.5% sucrose: *F*_(1, 15)_ = 0.256, *P* > 0.05; 4% sucrose: *F*_(1, 15)_ = 0.084, *P* > 0.05; 60% sucrose: *F*_(1, 13)_ = 1.629, *P* > 0.05]. A significant interaction between “pretreatment” and “time” was found in 60% sucrose responses [*F*_(5, 65)_ = 2.738, *P* < 0.05], analysis of simple effects revealed a marginal significant difference between the two pretreatment groups during the second 10 min [*F*_(1, 65)_ = 4.00, *P* = 0.067]. No significant interaction between “pretreatment” and “time” was found in 2.5% sucrose [*F*_(5, 75)_ = 1.699, *P* < 0.05] or 4% sucrose [*F*_(5, 75)_ = 0.840, *P* > 0.5]. Data analyses for the percentage of inactive nose-pokes showed no main effect of “pretreatment” [*F*_(1, 93)_ = 0.275, *P* > 0.05] or “concentration” [*F*_(5, 93)_ = 2.021, *P* > 0.05], as well as no significant interaction between “pretreatment” and “concentration” [*F*_(5, 93)_ = 0.754, *P* > 0.05] (the data was not shown), indicating that the differences in the number of reinforcers obtained could not be due to the changes of non-specific activity.

When rats were tested under a PR schedule on day 8, day 9, and day 10 of withdrawal (Figure 6), Two-Way repeated measures' ANOVA showed that only when responding for 60% sucrose did morphine rats earn more rewards than saline rats [*F*_(1, 13)_ = 16.154, *P* < 0.01]. No significant pretreatment effect was found when sucrose concentrations were under 60% [40%: *F*_(1, 16)_ = 1.660, *P* > 0.05; 30%: *F*_(1, 17)_ = 0.425, *P* > 0.05; 15%: *F*_(1, 17)_ = 0.136, *P* > 0.05; 4%: *F*_(1, 15)_ = 0.557, *P* > 0.05; 2.5%: *F*_(1, 15)_ = 0.229, *P* > 0.05]. As for the percentage of inactive nose-pokes (the data was not shown), there was a significant main effect of “concentration” [*F*_(5, 93)_ = 6.745, *P* < 0.001], but no significant main effect of “pretreatment” [*F*_(1, 93)_ = 0.013, *P* > 0.05] or interaction between “pretreatment” and “concentration” [*F*_(5, 93)_ = 1.091, *P* > 0.05].

### Effect of withdrawal on male copulatory behaviors

**Figure 7:**
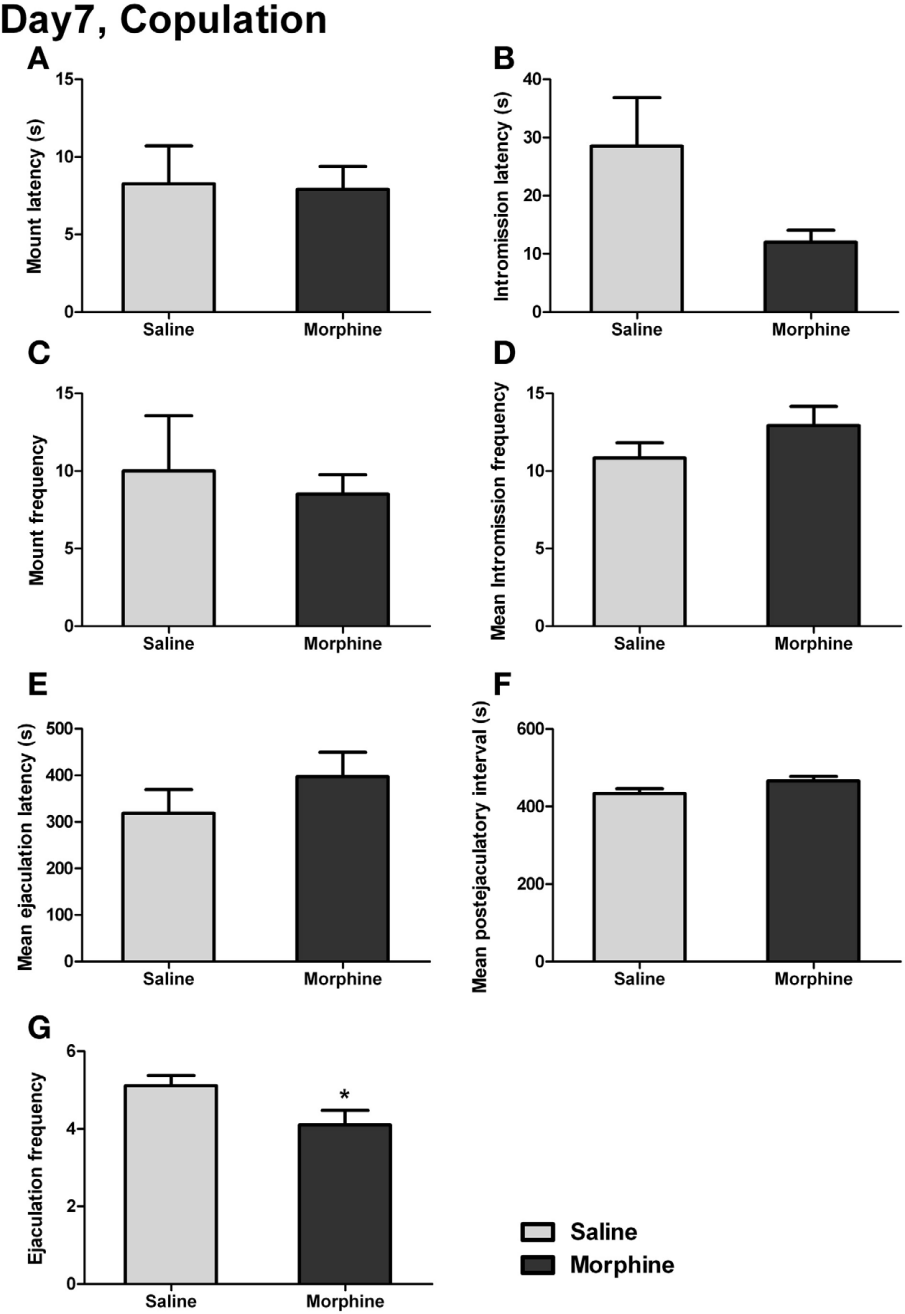
**The effects of morphine withdrawal on different copulatory measures in male rats**. The copulation testing was performed on day 7 of withdrawal. **(A)** Latency to first mount. **(B)** Latency to first intromission. **(C)** Number of mounts before the first ejaculation. **(D)** Mean number of intromissions prior to each ejaculation. **(E)** The mean of ejaculation latencies. **(F)** The mean of PEIs. **(G)** Total number of ejaculations. ^*^P < 0.05, relative to saline group. Values are mean ± s.e.m.

The male copulatory behaviors on day 7 of withdrawal were displayed in Figure 7. Withdrawal from morphine produced no significant effect on mount latency [Figure 7A: *t*_(16)_ = 0.13, *P* > 0.05] or intromission latency [Figure 7B: *t*_(15)_ = 2.03, *P* > 0.05], defined as the latency to the animal's first mount or intromission. No significant effect of withdrawal was found on either the mount frequency [Figure 7C: *t*_(17)_ = 0.41, *P* > 0.05], defined as the number of mounts before the first ejaculation, or the mean intromission frequency (IF) [Figure 7D: *t*_(17)_ = 1.30, *P* > 0.05], defined as the mean number of intromissions during the 1-h testing. Similarly, no significant difference in either the mean ejaculation latency [Figure 7E: *t*_(17)_ = 1.07, *P* > 0.05] or the mean postejaculatory interval (PEI) [Figure 7F: *t*_(17)_ = 1.85, *P* = 0.08] was found between saline-treated rats and morphine-treated rats. Nevertheless, a significant difference was noted for ejaculation frequency (EF) [Figure 7G: *t*_(17)_ = 2.15, *P* < 0.05], showing that the total number of ejaculations during the 1-h testing in morphine-treated rats was significantly decreased.

### Effect of withdrawal on simple appetitive behaviors

**Figure 8:**
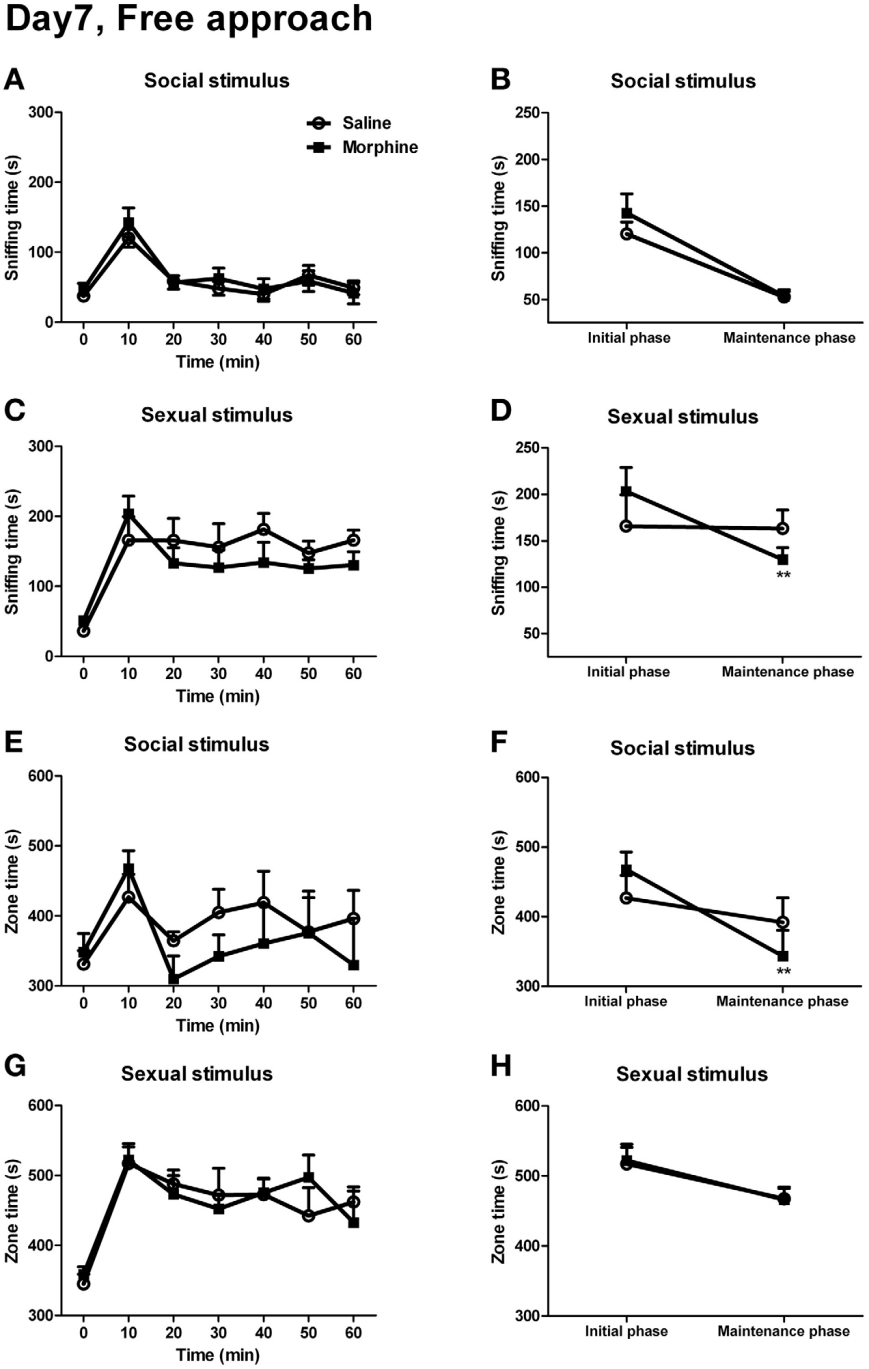
**The effects of morphine withdrawal on appetitive motivations for social or sexual reward in male rats under a free-approach condition (i.e., during the simple appetitive behavior testing)**. The 60-min testing (10 min/block) was performed on day 7 of withdrawal. **(A,C)** The time spent on sniffing the stimulus-cage holding a male or an estrous female rat. **(E,G)** The time spent in the incentive zone adjoined to the stimulus-cage holding a male or estrous female rat. **(B,D)** The sniffing time during the first 10-min block of the testing (initial phase) and the mean value of sniffing time during the last five 10-min blocks of the testing (maintenance phase) spent on the stimulus-cage holding a male or an estrous female rat. **(F,H)** The zone time during the initial phase of the testing and the mean value of zone time during the maintenance phase of the testing when the stimulus-cage held a male or an estrous female rat. Stars indicate a significant difference between maintenance phase and initial phase, ^**^P < 0.01. Values are mean ± s.e.m.

For the sniffing time and the zone time spent on social or sexual stimulus, no significant interaction between “time” and “pretreatment” was found [Figure 8A: *F*_(5, 8)_ = 0.353, *P* > 0.05; Figure 8C: *F*_(5, 9)_ = 2.261, *P* > 0.05; Figure 8E: *F*_(5, 8)_ = 1.432, *P* > 0.05; Figure 8G: *F*_(5, 10)_ = 0.881, *P* > 0.05]. However, between the two pretreatment groups, there was a visible dissociation in the sniffing time spent on sexual stimulus as well as the zone time spent on social stimulus. Furthermore, as it could be seen, the male subjects spent much time on sniffing the rewarding stimuli or spent much time in the incentive zone within the first 10 min of testing. Ten minutes later, the sniffing time and zone time decreased and remained relatively stable during the rest of testing. Hence, it seemed that the 1-h testing for the appetitive behaviors could be divided into two phases, the initial phase (the first 10 min of testing) and the maintenance phase (the remaining 50 min of testing). Thus, we converted the data during the maintenance phase into the averaged 10-min value to compare with the data during the initial phase. As expected, between “pretreatment” and “phase,” a significant interaction could be found for the sniffing time spent on sexual stimulus [Figure 8D: *F*_(1, 13)_ = 7.70, *P* < 0.05], and a near significant interaction was found for the zone time when the male stimulus was given [Figure 8F: *F*_(1, 12)_ = 3.51, *P* = 0.09]. The simple-effects analyses further revealed that, during from the initial phase to the maintenance phase, the sniffing time spent on sexual stimulus in morphine-treated rats showed a sharp decline [*F*_(1, 13)_ = 17.73, *P* < 0.01], while that in saline-treated rats remained almost unchanged [*F*_(1, 13)_ = 0.02, *P* > 0.05]. Similarly, for the zone time spent on social stimulus, there was a marked decrease in morphine-treated rats [*F*_(1, 12)_ = 18.53, *P* < 0.01] during the maintenance phase, while no significant decrease was found in drug-naïve rats [*F*_(1, 12)_ = 0.82, *P* > 0.05]. There was no significant interaction between “pretreatment” and “phase” for the sniffing time spent on social stimulus (Figure 8B) and the zone time spent on sexual stimulus (Figure 8H) (the statistics was not shown).

### Effect of withdrawal on decisional appetitive behaviors

**Figure 9:**
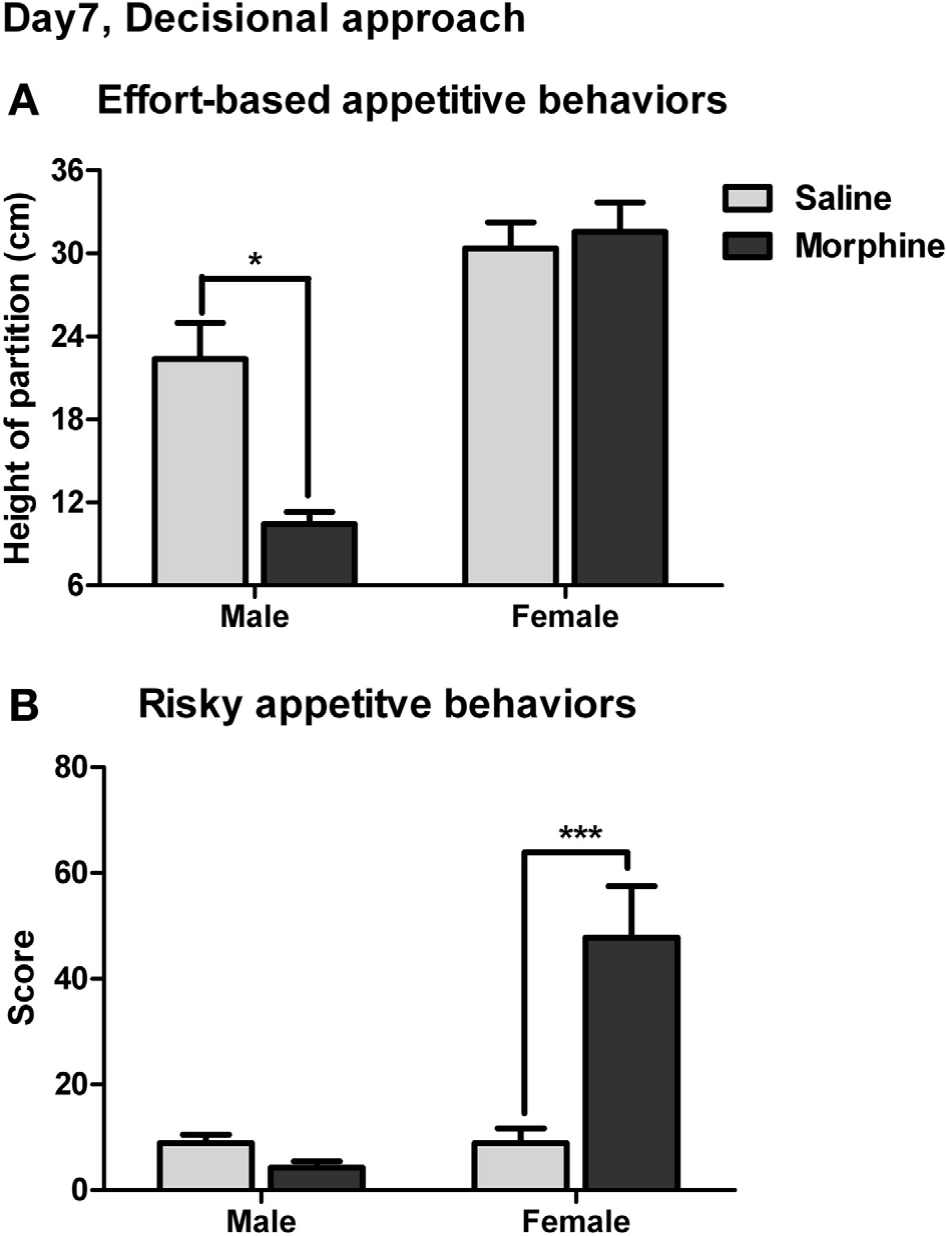
**The effects of morphine withdrawal on appetitive motivations for social or sexual reward in male rats under a restricted-approach condition (i.e., during the effort-based or risky appetitive behavior testing). The testing was performed on day 7 of withdrawal. (A)** The maximum height of the partition that animals were willing to climb over to approach the stimulus-cage holding a male or estrous female rat. **(B)** The total score for the amounts of difficulty that animals were willing to conquer (i.e., climb over a continuously heightened board thick with pins) to approach the stimulus-cage holding a male or estrous female rat. ^*^P < 0.05, ^***^P < 0.001, relative to saline group. Values are mean ± s.e.m.

The effort-based appetitive behaviors for the social and sexual rewards on day 7 of withdrawal were shown in Figure 9A. The main effect of “reward” was found [*F*_(1, 38)_ = 52.92, *P* < 0.001], demonstrating that the male subjects were always willing to expense more labors on approaching the sexual stimulus than on approaching the social stimulus. Moreover, a significant interaction was found between “pretreatment” and “reward” [*F*_(1, 38)_ = 10.82, *P* < 0.01]. The simple-effects analysis revealed a significant difference in the maximum height of the partition the subjects were willing to climb over to approach the social stimulus between saline and morphine groups [*F*_(1, 38)_ = 6.27, *P* < 0.05], but no significant difference was found between saline and morphine groups when the sexual stimulus was presented [*F*_(1, 38)_ = 0.00, *P* > 0.05]. These results suggested that after 7-days withdrawal, the morphine-exposed rats were unwilling to expense labors on approaching social stimulus compared to drug-naïve rats, but always made efforts to approach sexual stimulus, similar to drug-naïve rats.

The risky appetitive behaviors for the social and sexual rewards on day 7 of withdrawal were shown in Figure 9B. A significant interaction was found between “pretreatment” and “reward” [*F*_(1, 54)_ = 14.56, *P* < 0.001]. A simple-effects analysis further revealed that the score for the appetitive behavior for sexual stimulus in morphine-treated rats was remarkably higher than that in saline-treated rats [*F*_(1, 54)_ = 20.41, *P* < 0.001], while no significant difference in the score for the appetitive behavior for social stimulus was found between morphine and saline groups [*F*_(1, 54)_ = 0.24, *P* > 0.05]. Notably, in the drug-naïve animals, the score for the appetitive behavior for sexual stimulus was not significantly different from that for social stimulus [*F*_(1, 54)_ = 0.08, *P* > 0.05].

## Discussion

As is stated in the introduction, there exist large inconsistencies in the present preclinical literatures, i.e., the decreases (anhedonia-like), the increases (sensitization) or no change in hedonic response/motivation to natural stimuli following drug withdrawal (Lieblich et al., 1991; Barr et al., 1999; Barr and Phillips, 1999; Fiorino and Phillips, 1999; Hellemans et al., 2002; Russig et al., 2003; Cui et al., 2004; Nocjar and Panksepp, 2007; Zhang et al., 2007; Der-Avakian and Markou, 2010; Galaj et al., 2013). This is not surprising since the dosage/duration of drug pretreatment, withdrawal period, the type and magnitude of the supposed rewarding stimulus (e.g., the sweet pellet/liquid with different concentrations, sexual stimulus, or social stimulus), and the way and/or the amount of difficulty to get them may all play significant roles in the behavioral outputs. Therefore, the results derived from a specific experimental condition, particularly with limited parameters or testing conditions could only be used to verify the change of hedonic response or motivational state of animals in a certain situation. We consider this as one major source of the inconsistencies between different studies even with regard to basically the same scientific concerns (e.g., the motivational deficit after withdrawal from drugs of abuse). In this study, by usage of multiple rewarding stimuli and testing tasks, we managed to depicted a more clear and complete picture of motivated behaviors following short-term withdrawal from morphine.

In the sucrose consumption testing, morphine withdrawal reduced the intake of 2.5 and 4% sucrose solutions, but did not affect the consumption of sucrose solutions at higher concentrations (Figure 3). Obviously, the reduction in the intake of 2.5 or 4% sucrose solution was not due to the physical withdrawal symptoms, which had disappeared after 4 days of withdrawal (Figure 2B). And, considering the fact that the enormous weight loss caused by withdrawal might contribute to the amount of intake, the sucrose intake was calculated as a function of body weight in order to rule out this factor. Accordingly, the decreased consumption of 2.5 and 4% sucrose solutions may suggest a consummatory anhedonia for the sweet solution in animals withdrawn from morphine. Our findings corroborate previous reports (Lieblich et al., 1991; Hellemans et al., 2002) that opiate-withdrawn rats consume less of a sweet solution than do drug-naïve control subjects. However, in the present study, the manifestation of this consummatory anhedonia-like behavior critically depended on reward magnitude of the solutions (i.e., the sweetness of the sucrose solution), reflecting a deficit in the hedonic response only to the small reward and thus a probable reduction in appetite for such reward.

When reinforced with 15, 30, or 40% sucrose solution, the morphine-exposed animals performed fewer responses under a FR1 schedule (Figure 4), in accordance with the previous studies demonstrating that spontaneous opiate withdrawal (Harris and Aston-Jones, 2003) or naloxone-precipitated opiate withdrawal (Schulteis et al., 1994) decreased food-driven operant behavior under a FR schedule. Evidently, these diminished motivations to obtain the larger rewards could not be due to the withdrawal-induced deficit in hedonic response to or damaged appetite for the sucrose solution at 15, 30, or 40% concentration, since during the intake testing, the morphine-treated rats consumed as much the same solution as the saline-treated rats. Therefore, it was likely that the decrease in reinforcing efficacies of 15, 30, and 40% sucrose solutions following withdrawal resulted in the diminished motivation to work for the rewards in morphine-treated animals. Thus, it could be seen that the morphine-treated rats exhibited both consummatory anhedonia-like and motivational anhedonia-like behaviors for sucrose, but which were dissociably revealed depending on reward magnitude of the solutions as well as the testing tasks. Furthermore, the temporal analysis (Figure 5) showed that almost all the animals decreased their FR1 performance 10 min after the session started, but the morphine-exposed rats' responding for the 15, 30, and 40% sucrose solutions fell much faster, implying a trouble with the maintenance of the motivations.

Compared to the FR1 schedule, a PR schedule is more frequently used as a means to measure motivation (Markou et al., 1993; Brennan et al., 2001) or reinforcing efficacy of reward (Hodos, 1961; Hodos and Kalman, 1963; Richardson and Roberts, 1996) and more sensitive to changes in motivation (Barr and Phillips, 1998). Nevertheless, in contrast to the findings of anhedonia-like behaviors revealed by the FR1 schedule and the intake testing, a PR schedule in the present study failed to reveal any declined motivation to obtain the sucrose solution at the concentrations across from 2.5 to 40%, and even reflected an increased motivation for 60% sucrose solution in morphine-withdrawn animals (Figure 6). Actually, the results similar to ours have been reported by Phillips and Barr (Phillips and Barr, 1997), who found no change in PR performance following chronic mild stress (CMS), a well-validated animal model of depression, but did find the typical reduction in sucrose intake in the same animals. Our results are also consistent with the findings in the study carried out by Willner et al. (1998) and that study may help shed light on this paradoxical phenomenon. In that study, CMS did not change the performance under the PR schedule in rats reinforced with sugar-free pellets and even increased breaking point when the reward was sweet pellets (containing 10 or 90% sucrose). In addition, depressive mood induction does not change the PR performance maintained by the less palatable carob reinforcer, but similarly increased both chocolate-reinforced PR performance and chocolate craving in participants. And chocolate craving was significantly correlated with breakpoint in the PR schedule (Willner et al., 1998). Hence, it seems that performance under a PR schedule provides a measure of craving more than rewarding properties of the reinforcer and the increased PR performance is only observable under high incentive conditions. This point of view was further supported by our results that the unchanged and even increased PR performance existed simultaneously with the decreased or unchanged FR1 performance for the corresponding sucrose solutions. That is to say, in comparison with the FR schedule of reinforcement, under the PR procedure, a qualitatively different psychological construct, i.e., craving, which predominated over the rewarding efficacies of the sucrose solutions, may have decided the behavioral outputs of morphine-withdrawn animals. Thus, in the present study, we can see the heightened “craving” alongside with a consummatory anhedonia-like behavior and the motivational anhedonia-like behaviors in the morphine-withdrawn animals. And, besides reward magnitude, expression of these different behaviors in the same animals also heavily depends on usage of the testing tasks with different work requirements, i.e., the attenuated 2.5 and 4% sucrose consumptions under a free-access condition, a diminished motivation measured as the performance steadily reinforced by 15, 30, or 40% sucrose under a light work requirement (FR1 schedule) and the increased intensity of “craving” for 60% sucrose measured as the performance restrictedly reinforced by sucrose with a progressively increasing work requirement (PR schedule). Significantly, what makes this interpretation more convincing is that the behavioral patterns similar to those for sucrose can be seen in our following experiments on sexual and social rewards.

Sexual and social stimuli, as essential members of natural rewards, were used in the present study to examine the anhedonia-like behaviors, and some novel results were found with the modified behavioral procedure or the newly established behavioral models. The 1-h (but not 30-min) copulation test (Figure 7) showed that the male copulatory behaviors were slightly impaired in morphine-withdrawn animals, because a significant decrease was found only in ejaculation frequency (EF) without any significant change in other measures of sexual performance. Additionally, a near significant increase in PEI and a trend of increases in IF and ejaculatory latency (EL) were found. Since PEI is usually considered as an index of sexual motivation and, IF and EL may reflect the ejaculatory threshold (Ferrari and Giuliani, 1997), we think that the slight decrease in sexual appetite and ejaculatory dysfunction (i.e., the morphine-withdrawn rats might need more stimulation to ejaculate), although no significance was found, could have jointly contributed to the decrease in EF. Here we could observe a consummatory deficit in male sexual behavior on day 7 of withdrawal from morphine when the copulation test was prolonged to 1 h but not limited within 30 min, although the latter is widely used.

In the present study, the sexual and social motivations in animals were investigated under a free-approach condition or the decisional-approach conditions on day 7 of withdrawal from morphine. In the 1-h free-approach testing (simple appetitive behavior testing), the sexual or social appetitive motivation between saline-treated rats and morphine-treated rats displayed no significant difference at any time point of the testing (Figures 8A,C,E,G). Nevertheless, after the initial phase of the testing (the first 10 min), the saline-treated rats' motivations remained stable or only slightly decreased while the morphine-treated rats' motivations significantly declined (Figures 8D,F), indicating the trouble with maintenance of sexual and social motivations during withdrawal. Previous studies have shown that repeated exposure to drugs of abuse impairs sexual motivation during the earlier phase of withdrawal (Barr et al., 1999; Cui et al., 2004; Nocjar and Panksepp, 2007) but enhances sexual and social motivations after a long-term withdrawal (Fiorino and Phillips, 1999; Nocjar and Panksepp, 2002, 2007). Consistent with those studies, the present study found a mild increase in the sexual or social motivation during the first 10 min of the testing on day 7 of withdrawal which is probably a time point of transition from short-term withdrawal to long-term withdrawal. Yet, different from those studies which performed their motivation testing for 5 or 10 min, by extending the testing to 60 min, our study found a defect in maintenance of the motivations and a slightly lower sexual or social motivation in morphine-treated rats than in saline-treated rats during the maintenance phase. These results probably indicated the motivational anhedonia-like responses to the social and sexual stimuli in morphine-withdrawn animals. Furthermore, the motivational anhedonia-like response to the social stimulus in morphine-treated rats, although imperceptible under a free-approach condition, was clearly presented by the effort-based appetitive behavior testing.

In the effort-based appetitive behavior testing, the morphine-exposed rats showed a marked decrease in social motivation but no alteration in sexual motivation on day 7 of withdrawal (Figure 9A). Here, it is observable that the patterns for consummatory and motivational sexual behaviors in morphine-treated animals were exactly similar to their behavioral patterns for sucrose, i.e., the mildly impaired consummatory behaviors, the trouble with maintenance of motivated behaviors under a readily-reinforced or free-approach condition, and the unchanged performance under a PR or a PR-like procedure. Hence, it seems that the novel procedure used in the effort-based appetitive behavior test has successfully imitated the PR schedule in the operant behavior testing. Notably, for the high incentive reward, sexual reward, the increase of the effort-based appetitive behavior in morphine-withdrawn animals was absent and we think that this result was most probably due to a ceiling effect associated with the behavioral procedure under which the saline rats were also willing to consume efforts to approach the sexual stimulus. Consequently, based on this effort-related task, we established a PR-like procedure with increased amount of difficulty, i.e., with the aversive stimulus (needles), and discovered a remarkable increase in sexual appetitive motivation in morphine-withdrawn rats (Figure 9B). These results further support that the PR schedule or the PR-like procedure does reflect a “craving” (Willner et al., 1998), but not just reinforcing efficacy of the reward or a primary motivation to the rewarding stimulus, since the risky appetitive behavior testing was so difficult that the saline-treated rats displayed no higher motivation to approach the sexual stimulus than to approach the social stimulus. Thus, only a craving for sexual reward could drive animals to approach the sexual stimulus regardless of the progressively increased work as well as the aversive stimuli. Meanwhile, the present study suggests that whether or not the PR schedule can demonstrate an increased craving for rewards may depend not only on the incentive value of the reward (Willner et al., 1998) but also on the PR procedure itself.

In contrast to the behavioral pattern for sexual reward, the appetitive behavior for social reward in morphine-exposed rats was heavily depressed in the effort-based appetitive behavior testing, and was not different from that in saline-treated rats in the risky appetitive behavior testing (Figures 9A,B). It was unlikely that the decreased social appetitive behavior in drug-treated rats was due to the weight loss or weakened motor ability following withdrawal, since their sexual appetitive behaviors were not influenced by these factors at all. We think that the incentive value of the social vs. sexual stimuli may have decided the animals' behavioral outputs in the same testing tasks. The fact that the animals exhibited the lower motivations for social reward than for sexual reward both in the simple and the effort-based appetitive behavior tests (Figures 8, 9) confirmed that the social stimulus had a lower incentive value than the sexual stimulus. Hence, it was more likely that the morphine-treated rats were unwilling to expend labors on approaching a low-incentive reward, i.e., social stimulus. This is supported by the previous studies that found the decreased PR performance for a 4% sucrose solution (Zhang et al., 2007) or sucrose pellets (Der-Avakian and Markou, 2010) following drug withdrawal. It also appears to be one reason that the animals in those studies are tested in the absence of prior food deprivation, i.e., under a low incentive condition. In contrast, the animals in the present study were food and water deprived throughout all the operant tests, i.e., under the relatively higher incentive conditions, and thus displayed the unchanged or increased PR performance. Therefore, it might be suggested that a PR-like procedure could not only reflect a “craving” for the high-incentive reward, but also reveal the motivational anhedonia-like behavior when the animals were placed under a low incentive condition.

In summary, the present study depicted a multidimensional profile of anhedonia-like responses to multiple natural stimuli after about 1-week withdrawal from morphine. Different from the definite anhedonic responses elicited by acute withdrawal from drugs of abuse, we observed some quite complicated behaviors which vary based on reward magnitude (or incentive value) of rewarding stimulus and the type of testing task. The results are summarized in three points: (1) The anhedonia-like behavior was consistently found in animals withdrawn from morphine no matter which rewarding stimulus (sucrose, sexual stimulus, or social stimulus) was used. However, to each of the rewarding stimuli, the animals' anhedonia-like response could not be necessarily discovered if a single reward magnitude or a single testing task was adopted. Hence, we think that the anhedonia-like behavior has a characteristic of concealment once after an acute withdrawal period, as the results having shown. For example, only the sucrose solution at a low concentration (2.5 or 4%) could reflect an anhedonia-like behavior during the sucrose intake testing. A significant decrease in EF was revealed only if the copulation testing was prolonged to 1 h. The decreased motivation to the natural rewards became manifest until the maintenance phase of the simple appetitive behavior testing as well as the FR1 performance. (2) The present study found a dissociation of motivational anhedonia from consummatory anhedonia, which was evidenced by that the morphine-withdrawn animals did not display an anhedonia-like response to 15, 30, or 40% sucrose solution during the intake testing, but showed a diminished motivation to work for the same sucrose solution measured by the FR1 performance. This is similar to some extent to the dissociation noted in the clinical literature which mentions that many patients with anhedonia appear to enjoy rewards that were readily available, yet complained about feeling no desire to obtain them (Treadway and Zald, 2011). (3) The anhedonia-like behaviors coexisted with heightened craving for the high-incentive reward following morphine withdrawal, i.e., despite the consummatory and motivational anhedonia-like responses to multiple rewarding stimuli, the PR responding for 60% sucrose solution and the risky appetitive behavior for sexual stimulus was markedly increased. The risky appetitive behavior testing is not only an effort-based but also a risk-related task since the presence of the aversive or dangerous stimulus. Obviously, in contrast with the inhibited sexual appetitive behavior in saline-treated animals, the repeated exposure to morphine released it from inhibitory control. This result is consistent with the notion that one of the largest and most problematic effects of drugs on sexual behavior in human is the increase in risk-taking behaviors related to sexual activity (Frohmader et al., 2010). More significantly, these extremely high-motivated and risk-taking behaviors took place only when the animals were facing the high-incentive reward (i.e., high concentration of sucrose and sexual reward). Thus, it could be believable that the craving for various abused drugs which are the highly incentive-sensitized stimuli, alongside with the impaired inhibitory control would form one of the psychological mechanisms underlying drug relapse.

## Author contributions

Xigeng Zheng conceived the research project, designed the experiments, interpreted the results and revised the manuscript. Yingying Li performed the experiments for sucrose and withdrawal syndromes, analyzed and interpreted the related data. Yunjing Bai performed the experiments for social and sexual rewards, analyzed and interpreted the related data, drafted the manuscript. Yaodi Lv assisted with performance of the experiments for social and sexual rewards. Zhengkui Liu provided necessary support for the accomplishment of our experiments.

### Conflict of interest statement

The authors declare that the research was conducted in the absence of any commercial or financial relationships that could be construed as a potential conflict of interest.
